# Cerebellar transcriptional alterations with Purkinje cell dysfunction and loss in mice lacking PGC-1α

**DOI:** 10.3389/fncel.2014.00441

**Published:** 2015-01-06

**Authors:** Elizabeth K. Lucas, Courtney S. Reid, Laura J. McMeekin, Sarah E. Dougherty, Candace L. Floyd, Rita M. Cowell

**Affiliations:** ^1^Department of Psychiatry and Behavioral Neurobiology, University of Alabama at BirminghamBirmingham, AL, USA; ^2^Department of Neuroscience, Icahn School of Medicine at Mount SinaiNew York, NY, USA; ^3^Department of Neuroscience, Johns Hopkins University School of MedicineBaltimore, MD, USA; ^4^Department of Physical Medicine and Rehabilitation, University of Alabama at BirminghamBirmingham, AL, USA

**Keywords:** PPARGC1A, cerebellum, ataxia, Catwalk, stereology, Refsum disease, Friedreich Ataxia

## Abstract

Alterations in the expression and activity of the transcriptional coactivator peroxisome proliferator-activated receptor γ coactivator-1α (*ppargc1a* or PGC-1α) have been reported in multiple movement disorders, yet it is unclear how a lack of PGC-1α impacts transcription and function of the cerebellum, a region with high PGC-1α expression. We show here that mice lacking PGC-1α exhibit ataxia in addition to the previously described deficits in motor coordination. Using q-RT-PCR in cerebellar homogenates from PGC-1α^−/−^ mice, we measured expression of 37 microarray-identified transcripts upregulated by PGC-1α in SH-SY5Y neuroblastoma cells with neuroanatomical overlap with PGC-1α or parvalbumin (PV), a calcium buffer highly expressed by Purkinje cells. We found significant reductions in transcripts with synaptic (complexin1, Cplx1; Pacsin2), structural (neurofilament heavy chain, Nefh), and metabolic (isocitrate dehydrogenase 3a, Idh3a; neutral cholesterol ester hydrolase 1, Nceh1; pyruvate dehydrogenase alpha 1, Pdha1; phytanoyl-CoA hydroxylase, Phyh; ubiquinol-cytochrome c reductase, Rieske iron-sulfur polypeptide 1, Uqcrfs1) functions. Using conditional deletion of PGC-1α in PV-positive neurons, we determined that 50% of PGC-1α expression and a reduction in a subset of these transcripts could be explained by its concentration in PV-positive neuronal populations in the cerbellum. To determine whether there were functional consequences associated with these changes, we conducted stereological counts and spike rate analysis in Purkinje cells, a cell type rich in PV, from PGC-1α^−/−^ mice. We observed a significant loss of Purkinje cells by 6 weeks of age, and the remaining Purkinje cells exhibited a 50% reduction in spike rate. Together, these data highlight the complexity of PGC-1α's actions in the central nervous system and suggest that dysfunction in multiple cell types contribute to motor deficits in the context of PGC-1α deficiency.

## Introduction

Since the identification of peroxisome proliferator activated receptor γ coactivator-1α (PGC-1α) as a master regulator of mitochondrial biogenesis in brown adipose tissue (Puigserver et al., 1998), a number of studies have demonstrated an association between PGC-1α dysfunction and neurological disorders, including Huntington Disease (Cui et al., 2006; Weydt et al., 2006; Chaturvedi et al., 2010; Hathorn et al., 2011; Johri et al., 2012; Puddifoot et al., 2012; Soyal et al., 2012), Parkinson Disease (St-Pierre et al., 2006; Zheng et al., 2010; Clark et al., 2011; Shin et al., 2011; Thomas et al., 2012), and Alzheimer Disease (Qin et al., 2009; Sheng et al., 2012; Pedros et al., 2014). Germline (Lin et al., 2004; Leone et al., 2005; Lucas et al., 2012) and nervous system-specific (Lucas et al., 2012) deletion of PGC-1α causes pronounced neurodegeneration in brain regions involved in motor control and deficits in motor coordination, and PGC-1α overexpression can promote neuronal survival and function in some mouse models (Cui et al., 2006; Mudo et al., 2012; Tsunemi et al., 2012). However, little is known about the physiological impact of PGC-1α dysfunction on specific cell types and circuits in the brain.

PGC-1α can interact with a number of transcription factors and coactivators to drive expression of metabolic transcriptional programs in peripheral tissues (Lin, 2009). Throughout the brain, PGC-1α protein is highly concentrated in neurons expressing the enzyme glutamic acid decarboxylase 67 (GAD67; Cowell et al., 2007). We recently determined that in GAD67-positive interneurons of the cortex, PGC-1α is involved in regulating the calcium buffer parvalbumin (PV), the synaptic proteins synaptogamin 2 (Syt2) and complexin 1 (Cplx1), and the structural protein neurofilament heavy chain (Nefh; Lucas et al., 2010, 2014). These data indicate that PGC-1α can drive genes besides those involved in metabolic regulation and that PGC-1α's role in neurons may be more complex than previously appreciated.

Considering the high concentration of PGC-1α in the cerebellum and the role of the cerebellum in motor coordination, we hypothesized that mice lacking PGC-1α would show transcriptional deficits and evidence of cerebellar dysfunction. Here we report that PGC-1α^−/−^ mice exhibit ataxia and reductions in genes involved in metabolism, synaptic function, and structural support. Alterations in protein expression are restricted to specific neuronal populations within the cerebellum, especially Purkinje cells, and Purkinje cell number and firing rate are decreased in PGC-1α^−/−^ animals. These experiments suggest that PGC-1α is required for proper gene expression in the cerebellum and that cerebellar deficits contribute to motor abnormalities associated with PGC-1α deficiency.

## Methods

### Animals

All experimental protocols were approved by the Institutional Animal Care and Use Committee of the University of Alabama at Birmingham. PGC-1α^−/−^ mice (Lin et al., 2004) were used for experiments, and PGC-1α^+/+^, ^+/−^, and ^−/−^ mice were obtained from offspring of PGC-1α^+/−^ breeding pairs. Conditional deletion of PGC-1α was produced by crossing mice with LoxP sites flanking the exon 3–5 region of the PGC-1α gene (Lin et al., 2004; gift of Bruce Spiegelman, Harvard University, Cambridge, USA) with mice expressing Cre recombinase driven by the PV promoter (Hippenmeyer et al., 2005; #008069 from the Jackson Laboratory, Bar Harbor, ME, USA). For conditional knockout experiments, littermates expressing Cre recombinase without loxP sites were used as controls. To determine specificity of the Cre-mediated recombination pattern, PV-Cre mice were crossbred to mutant tomato/mutant green reporter mice (Muzumdar et al., 2007; #007676 from Jackson Laboratory). All mice were maintained on a C57BL6/J genetic background and housed 2–5 in a cage at 26 ± 2°C room temperature with food and water *ad libitum*. With the exception of behavioral analysis, experiments were conducted on male and female mice, and no significant differences were found between male and female mice in any measure.

### CatWalk gait analysis

Gait analysis was performed on 6-month-old male PGC-1α^−/−^ mice with the CatWalk system (Noldus Information Technology, Leesburg, VA) as previously described (Hamers et al., 2006). Briefly, mice traversed a glass walkway (109 × 15 × 0.6 cm) with dark plastic walls spaced 15 cm apart in a dark room. Light from an encased fluorescent bulb was internally reflected within the glass walkway and scattered when the plantar surface of the paw contacted the walkway floor, thereby producing paw prints. Paw prints were recorded by a high-speed CCD camera mounted below the walkway at 50 half-frames/s and were stored on a computer by the associated CatWalk 7.1 acquisition software. Trials in which the animal stopped or changed direction were excluded from subsequent analysis, and three uninterrupted trials were analyzed and averaged to obtain the final gait analysis values. Paw print designations were assigned and data analyzed using the CatWalk analysis software (v 7.1) by an experimenter who was blinded to the genotype of the animals. Mice were tested at 6 months of age because PGC-1α^−/−^ animals weighed significantly less than PGC-1α^+/+^ animals at earlier time points (data not shown), which could bias Catwalk detection and subsequent analyses of paw prints.

### Gene expression analyses

Mice, aged 1 month for germline and 6 months for conditional PGC-1α deletion, were anesthetized with isoflurane before sacrifice by decapitation. Cerebella were collected in centrifuge tubes, flash frozen on dry ice, and stored at −80°C. Before processing, samples were incubated in RNA*later*®-ICE (Ambion, Austin, TX, USA) according to manufacturer's instructions. Tissue was homogenized with Tissue-Tearor (Biospec, Bartlesville, OK, USA) in Trizol reagent, and RNA was isolated by the Trizol/choloform-isopropanol method following the manufacturer's instructions (Invitrogen, Carlsbad, CA, USA). RNA concentrations and purity were quantified using a Thermo Scientific NanoDrop2000 (Fisher Scientific, Pittsburg, PA, USA). Equivalent amounts of RNA (1 μg) were treated with DNase I (Promega, Madison, WI, USA) at 37°C for 30 min, and DNase was inactivated at 65°C for 15 min. RNA was reverse-transcribed using the High-Capacity cDNA Archive Kit (Applied Biosystems, Carlsbad, CA, USA). Taqman qPCR was performed with JumpStart Taq Readymix (Sigma, St. Louis, MO, USA) and the following mouse-specific Applied Biosystems primers listed in Supplementary Table 1.

Reaction protocols consisted of an initial ramp time (2 min, 50°C; 10 min, 95°C) and 40 subsequent cycles (15 s, 95°C; 1 min, 60°C). Relative concentrations of the genes of interest were calculated in comparison to a standard curve calculated from dilutions of cDNA (1:5, 1:10, 1:20) from a pooled sample of wildtype littermate controls. Values were normalized to β-actin (ABI# Mm00607939_s1) or 18S rRNA (ABI# Hs99999901_s1) for values for the same sample and then expressed as ratio to control samples ± SEM.

### Western blot analysis

Primary antibodies included rabbit anti-Cplx1,2 (Synaptic Systems, Goettingen, Germany), chicken anti-Nefh (Abcam, Cambridge, MA, USA), rabbit anti-pacsin2 (Sigma), and mouse anti-β-actin (Chemicon, Billerica, MA, USA). One-month-old PGC-1α^+/+^ and ^−/−^ mice were anesthetized with isoflurane before sacrifice by decapitation. Cerebella were collected in centrifuge tubes, flash frozen on dry ice, and stored at −80°C. Cerebella were homogenized in RIPA buffer (150 mM NaCl, 50 mM Tris, 1% Triton X-100, 1% sodium dodecyl sulfate, 0.5% deoxycholic acid; pH 8.0) containing a protease inhibitor tablet. Total protein concentration was determined with a bicinchonicic acid protein assay kit (Thermo Scientific, Waltham, MA, USA), and absorbance was measured at 540 nm. Protein was denatured in sample buffer (62.5 mM Tris-HCl, 20% glycerol, 2% sodium dodecyl sulfate, 5% β-mercaptoethanol; pH 6.8) at 95°C. Equivalent amounts of protein were loaded into precast polyacrylamide NuPage gels (Invitrogen). One interblot control sample was loaded onto every gel to permit comparison among gels. Protein was transferred onto nitrocellulose membranes. Membranes were blocked with 5% milk in tris buffered saline (TBS; pH 7.6) with 1% Tween (TBS-T) and probed with primary antibodies in 5% IgG-free bovine serum albumin (BSA; Jackson ImmunoResearch, West Grove, PA, USA) TBS-T overnight at 4°C and peroxidase-conjugated secondary antibodies (Jackson ImmunoResearch) in 5% milk TBS-T for 1 h at room temperature. Membranes were incubated in chemiluminescent substrate (Thermo Scientific) and exposed to film. The optical density of bands was calculated after background subtraction using UN-SCAN-IT gel analysis software (Silk Scientific Inc., Orem, UT, USA). All bands were normalized to the interblot control band, then to actin, and expressed as optical density mean ± SEM.

### Immunofluorescence

Animals were anesthetized with isoflurane and perfused intracardially with phosphate-buffered saline (PBS, pH 7.4) and 4% paraformaldehyde in PBS. Brains were removed, postfixed in 4% paraformaldehyde for 24–72 h, cryoprotected in graded sucrose (5–20%), embedded in a mixture of 20% sucrose and Tissue-Tek O.C.T. Compound (Sakura Finetek, Torrance, CA), and frozen at −80°C. Tissue blocks were sectioned at 20 μm, mounted onto charged slides (Fisher, Hampton, NH), and allowed to dry overnight before freezing at −80°C.

The same primary antibodies were used for immunofluorescence as Western blotting (see above) with the addition of mouse anti-GAD67 (Chemicon), mouse anti-PV (Sigma), and rabbit anti-PV (Swant, Marly, Switzerland). Slides were thawed, washed in PBS, and blocked with 10% serum from the host of the secondary antibody in PBS for 1 h. Sections were then incubated in the primary antibody overnight with 3% BSA and 5% serum from the host of the secondary antibody in PBS with Triton-X100 (PBS-T; Sigma) at 4°C. Slides were washed in PBS-T and PBS and incubated 2 h at room temperature with the corresponding fluorescence-conjugated secondary antibody (Jackson ImmunoResearch) with 5% serum for the host of the secondary antibody in PBS-T. For colabeling with GAD67, a mouse-on-mouse kit (Vector Laboratories, Burlingame, CA) was used following the manufacturer's instructions to reduce background staining. Sections were coverslipped with Prolong Antifade Gold (Invitrogen) and stored at 4°C. All images were captured with a Leica confocal microscope (Buffalo Grove, IL, USA). All confocal settings, including laser intensity, gain, offset, and zoom, were held constant across all groups for a given protein. Images were imported into Adobe Photoshop CS3 (Adobe, San Jose, CA) for adjustments to contrast and brightness.

### Stereology

Animals were transcardially perfused, and brains were processed as described above for immunofluorescence. Brains were sectioned 30 μm thick in the sagittal plane and stored at −80°C until use. Hematoxylin and Eosin (H&E; Sigma) staining was conducted according to the manufacturer's instructions on serial cerebellum sections to visualize Purkinje cells. Stereological analyses were performed with a computerized stereology workstation, consisting of a modified Olympus BX51 light microscope (Center Valley, PA, USA) equipped with 4×–100× Plan Apo objectives, a motorized specimen stage (Ludl Electronic Products Ltd., Hawthorne, NY, USA), CCD color video camera (CX9000, Microfire Optronics, Goleta, CA, USA) and StereoInvestigator 10 software (MBF Biosciences, Williston, VT, USA). The optical fractionator principle (West et al., 1991) was used for estimation of Purkinje cells in the cerebellum. Systematic random sampling in every 20th section was performed in a total of 11 sections per animal. All cells whose nucleus top came into focus within the unbiased counting spaces throughout the delineated regions were counted. The systemic random sampling grid layout was 253 × 188 μm with 100 desired sites per animal. A 60 × 60 μm square dissector frame was used to sample the sections.

### Loose patch recordings

Loose patch recordings of Purkinje cells were conducted as in Dougherty et al. (2012). Mice (P42 ± 5 days) were anesthetized with isoflourane and then killed by decapitation. Brains were places in ice-cold artificial cerebral spinal fluid (ACSF) containing the following (in mM): 125 NaCl, 2.5 KCl, 2 CaCl_2_, 1 MgCl_2_, 25 NaHCO_3_ 1.25 Na_2_HPO_4_and 25 D-glucose with a pH of 7.4 and osmolality of 295 ± 5 mOsm. ACSF was bubbled with 95%O_2_/5%CO_2_. Sagittal cerebellar slices (300 μm thick) were cut using a Vibratome (7000 smz, Campden Instruments, Sarasota, FL, USA). The slices were allowed to rest for 60 min at room temperature (22–23°C), and all subsequent procedures were performed at room temperature. Slices were superfused continuously with oxygenated recording ACSF at room temperature. Slices were viewed with an upright microscope (Zeiss Axio Examiner A1, Thornwood, NY, USA) using infrared-differential interference contrast optics. Loose patch recordings were acquired from visually identified Purkinje cells using Axio Vision 4.8 software (Zeiss). Cellular activity was recorded using internal solution containing the following (in mM): 140 K-gluconate, 1 EGTA, 10 HEPES and 5 KCL, pH 7.3. Pipette tip resistance was 2–5 MΩ. The extracellular recording pipettes containing the internal solution were placed under visual control directly adjacent to the soma of the cell of interest. Positive pressure was applied throughout this process followed by a brief release of pressure to form a seal averaging approximately 45 MΩ. Cell-attached, loose patch-clamp recordings were obtained using an Axon CNS Molecular Devices amplifier (Multiclamp 700B, Molecular Devices, Sunnydale, CA, USA), filtered at 10 kHz and digitized at 20 kHz (Digidata 1440A, Molecular Devices). A 3 min gap-free protocol on Clampex 10.2 software (Molecular Devices) was used for data collection. Detection and analysis of event frequency and inter-event interval were performed semi-automatically using the program Clampfit 10.2. The detection threshold was set for analysis based on the event amplitude from a given cell.

### Statistical analyses

All statistical analyses were conducted with SPSS software (IBM, Armonk, NY, USA). For initial gene expression studies, One-way ANOVA comparing ^+/+^, ^+/−^, and ^−/−^ mice followed by Fisher's LSD was implemented. After determination of putative gene targets in the cerebellum, confirmation of protein expression in ^−/−^ mice and gene expression in conditional knockouts was analyzed with one-tailed *t*-tests with the *a priori* hypothesis that gene and protein expression would decrease in mice lacking PGC-1α. All other differences between PGC-1α^+/+^ and ^−/−^ mice were detected by two-tailed *t*-tests. All data are presented as mean ± SEM.

## Results

### Mice lacking PGC-1α exhibit ataxia

**Figure 1:**
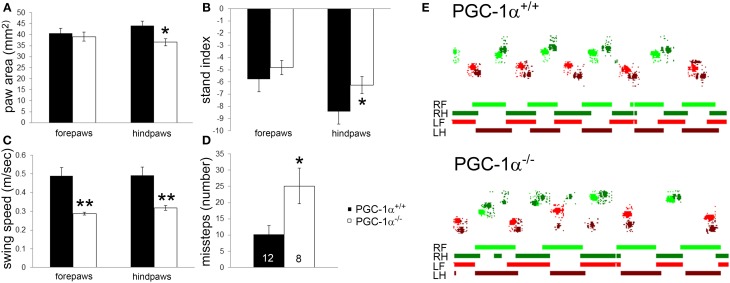
**Ataxia characterized by altered gait kinematics in mice lacking PGC-1α**. CatWalk gait analysis was performed on male PGC-1α^+/+^ and ^−/−^ littermates at 6 months of age. PGC-1α^−/−^ mice exhibited decreased hindpaw area **(A)**, increased hindpaw stand index **(B)**, decreased forepaw and hindpaw swing speed **(C)**, and an increased number of missteps **(D)** compared to their littermate controls. Representative digitized paw prints and associated step cycles are shown in **(E)** with RF, right front paw, RH, right hind paw, LF, left front paw, and LH, left hind paw. Two-tailed *t*-tests. ^*^p < 0.05, ^**^p < 0.005. n/group indicated on last bar histogram. Data are presented as mean ± SEM.

PGC-1α^−/−^ mice were previously described to have severe motor abnormalities indicative of neurological dysfunction, such as impaired rotarod performance, decreased rearing behavior, hindlimb clasping, and tremor (Lucas et al., 2012). Given the high expression of PGC-1α in the cerebellum compared to other brain regions (Cowell et al., 2007), we hypothesized that motor impairment in PGC-1α^−/−^ animals would include ataxia and conducted CatWalk gait analyses on male PGC-1α^+/+^ and ^−/−^ littermates at 6 months of age. CatWalk gait analyses have been validated as indicators of motor abnormalities in rodents, and several CatWalk variables indicating paw print dimensions and the time and distance relationships between footfalls are affected in rodent models of neurological diseases in which PGC-1α has been implicated, such as Huntington (Vandeputte et al., 2010) and Parkinson (Vlamings et al., 2007; Chuang et al., 2010) Diseases. We assessed paw print area, which indicates the average area of each paw print over a 4 step cycle sequence, and found that PGC-1α^−/−^ mice exhibited reduced hindpaw area compared to PGC-1α^+/+^ mice [*t*_(18)_ = 2.53, *p* = 0.02; Figure 1A]. We also evaluated the stand index, which is ratio of all maximum paw contact values (stance) over the stance duration (seconds), normalized for camera acquisition rate. PGC-1α^−/−^ animals exhibited an increased hindpaw stand index [*t*_(18)_ = 2.53, *p* = 0.02], indicating greater duration stance than PGC-1α^+/+^ mice (Figure 1B). Similarly, the average speed in the swing phase of the step cycle, or swing speed, was found to be decreased for the forepaws [*t*_(18)_ = 3.74, *p* = 0.002] and hindpaws [*t*_(18)_ = 3.25, *p* = 0.004] of PGC-1α^−/−^ mice (Figure 1C). Missteps indicate an instance in the step cycle wherein a paw was not placed in a step sequence, and PGC-1α^−/−^ mice exhibited an increased number of missteps compared to their wildtype littermates [*t*_(15)_ = 2.58, *p* = 0.02; Figure 1D]. Figure 1E is comprised of representative traces showing color-coded, digitized paw prints and corresponding step cycles for PGC-1α^−/−^ and PGC-1α^+/+^ mice. Taken together, these data demonstrate that the motor phenotype of PGC-1α^−/−^ mice includes ataxia characterized by altered gait kinematics, including increased stance duration, increased paw placement, and stepping mistakes.

### Novel cerebellar PGC-1α-dependent genes

Little is known about the downstream gene targets of PGC-1α in the cerebellum, despite the high concentration of PGC-1α in this brain region (Cowell et al., 2007). To identify novel PGC-1α-dependent transcripts in the cerebellum, we used an approach our laboratory recently used to identify PGC-1α-dependent genes in the cortex in which we mined microarray data comparing human SH-SY5Y neuroblastoma cells overexpressing PGC-1α and GFP in tandem to cells expressing GFP alone (Lucas et al., 2014; GEO NCBI database registration in progress). Transcripts were selected to measure in cerebellar homogenates based on three criteria: (1) all of the top 10 transcripts significantly upregulated by PGC-1α overexpression with a murine homolog, (2) transcripts listed on the whole-brain PGC-1α Neuroblast feature from the Allen Brain Atlas, and (3) genes listed on the whole-brain parvalbumin (PV) Neuroblast feature from the Allen Brain Atlas. The Neuroblast feature of the Allen Brain Atlas identifies genes with similar 3D spatial expression profiles by conducting Pearson's correlation coefficients of the expression intensity of each gene with other genes coexpressed in 200 μm cubes (Lein et al., 2007). Although PGC-1α is not required for the expression of PV in the cerebellum as it is in the forebrain (Lucas et al., 2010), the PV Neuroblast was implemented because of the significant overlap between the neuroanatomical localization of PV and PGC-1α in this brain region; transcript and protein expression of both PGC-1α and PV is enriched Purkinje cells, molecular layer interneurons, and neurons of the deep cerebellar nuclei (Celio, 1990; Cowell et al., 2007). Mined transcripts fitting our three criteria but with unknown functions or ubiquitous expression patterns when viewed on the Allen Brain Atlas were excluded from subsequent analysis (Supplementary Table 2). The final list consisted of 37 transcripts (Supplementary Table 3).

**Figure 2:**
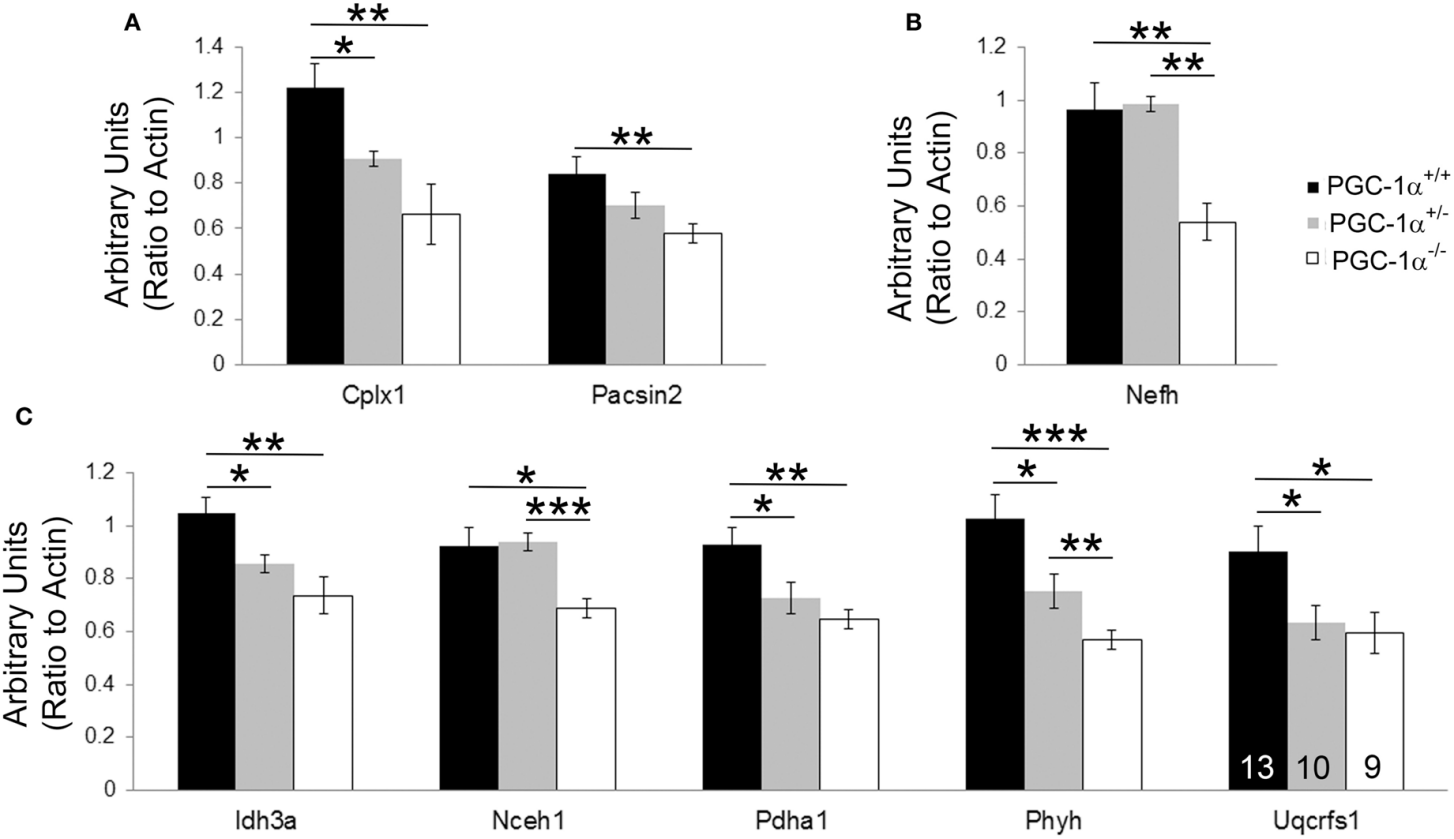
**Novel cerebellar PGC-1α-dependent genes**. q-RT-PCR of microarray-identified PGC-1α regulated transcripts was performed on cerebellar homogenates from PGC-1α^+/+^, ^+/−^, and ^−/−^ littermates at 30 days of age. Expression of transcripts spanning synaptic (Cplx1, Pacsin2; **(A)**, structural [Nefh; **(B)**], and metabolic [Idh3a, Nceh1, Pdha1, Phyh, Uqcrfs1; **(C)**] functions were significantly decreased in PGC-1α^−/−^ compared to littermate control cerebella. One-Way ANOVA followed by Fisher's LSD. ^*^p < 0.05, ^**^p < 0.005, ^***^p < 0.0005. n/group indicated on last bar histogram. Data are presented as mean ± SEM.

Expression levels of all 37 transcripts were measured, and eight transcripts were found to be significantly reduced in cerebellar homogenates from PGC-1α^−/−^ animals compared to their littermate controls at 30 days of age (Figure 2). These included the synaptic transcripts complexin 1 [Cplx1; *F*_(2, 14)_ = 8.84, *p* = 0.005] and pacsin2 [*F*_(2, 31)_ = 4.66, *p* = 0.02], the structural transcript neurofilament heavy chain [Nefh; *F*_(2, 15)_ = 11.87, *p* = 0.001], and the metabolism-related transcripts isocitrate dehydrogenase 3a [Idh3a; *F*_(2, 15)_ = 8.75, *p* = 0.004], neutral cholesterol ester hydrolase 1 [Nceh1; *F*_(2, 31)_ = 6.42, *p* = 0.005], pyruvate dehydrogenase alpha 1 [Pdha1; *F*_(2, 31)_ = 6.77, *p* = 0.004], phytanoyl-CoA hydroxylase [Phyh; *F*_(2, 31)_ = 11.46, *p* = 0.0002], and ubiquinol-cytochrome c reductase, Rieske iron-sulfur polypeptide 1 [Uqcrfs1; *F*_(2, 31)_ = 4.47, *p* = 0.02]. Phyh was the only gene to exhibit a dose-dependency with PGC-1α expression, with significant differences between all genotypes, while deletion of one allele was sufficient to cause decreases in Cplx1, Idh3a, Pdha1, and Uqcrfs1 (Fisher's LSD, *p* < 0.05).

### Neuroanatomical overlap of PGC-1a with novel PGC-1α-dependent genes in the mouse cerebellum

**Figure 3:**
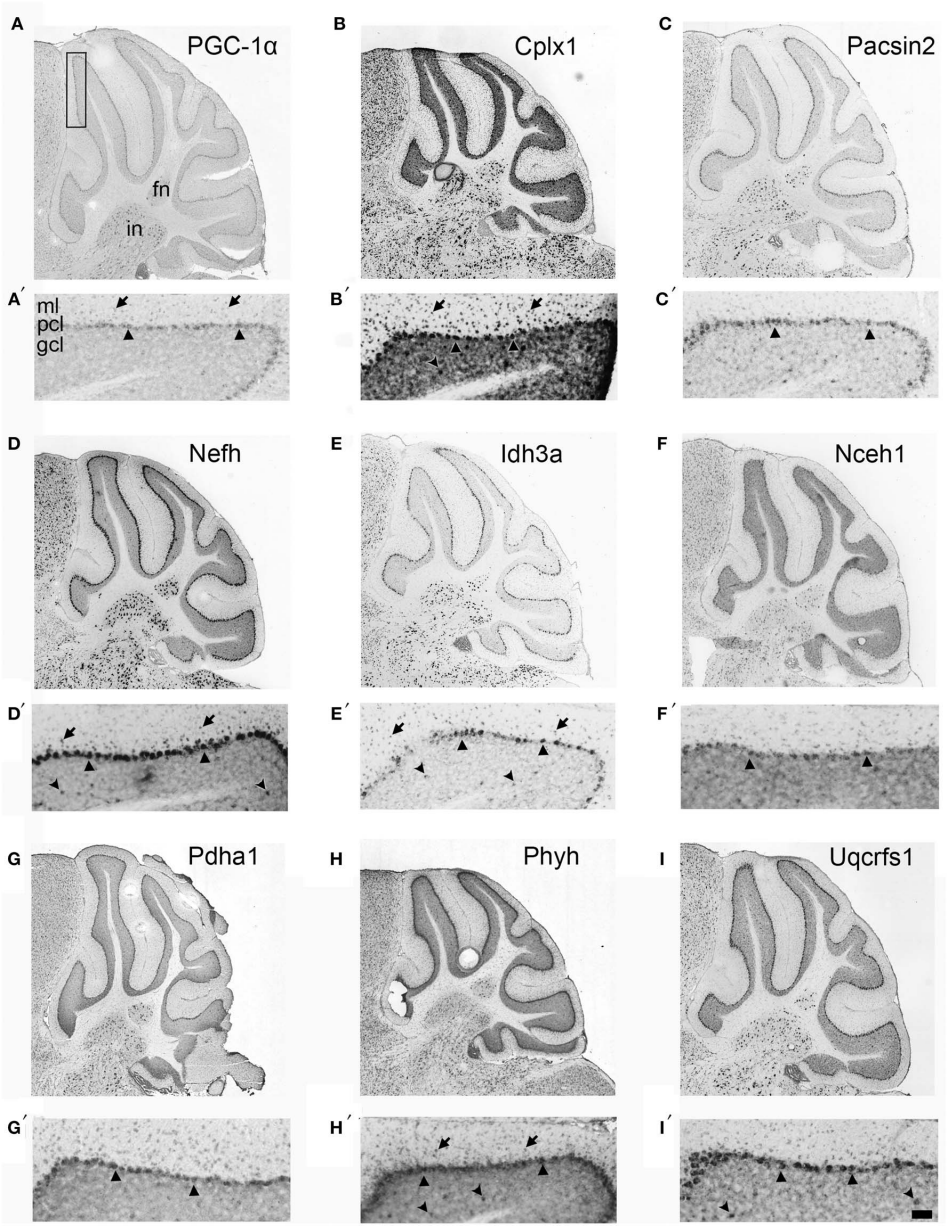
**Neuroanatomical mRNA localization of PGC-1α and its dependent genes in the cerebellum**. Sagittal *in situ* hybridization images of PGC-1α **(A)**, Cplx1 **(B)**, Pacsin2 **(C)**, Nefh **(D)**, Idh3a **(E)**, Nceh1 **(F)**, Pdha1 **(G)**, Phyh **(H)**, and Uqcrfs1 **(I)** transcript expression in the adult mouse cerebellum were collected from the Allen Brain Atlas (http://www.brain-map.org). All transcripts are highly expressed in the Purkinje cell layer (PCL; arrowheads) and the fastigial (FN) and interposed (IN) deep cerebellar nuclei. Most transcripts also exhibited expression in the molecular layer (ML; arrows), and Cplx1, Nefh, Phyh mRNA exhibited expression in the granule cell layer (GCL; concave arrowheads). Boxed portion in A represents area of higher magnification images in **A′–I′**. Scale bar = 525 μm for **A–I** and 100 μm for **A′–F′**.

If Cplx1, Pacsin2, Nefh, Idh3a, Nceh1, Pdha1, Phyh, and Uqcrfs1 are truly PGC-1α-dependent genes, we surmised that they would exhibit neuroanatomical overlap with PGC-1α in the cerebellum. With the exception of Cplx1, Pacsin2, and Nefh, antibodies against the protein products of PGC-1α-dependent transcripts are either not commercially available or do not work well for immunohistochemistry. Therefore, we collected *in situ* hybridization images published on the Allen Brain Atlas (http://www.brain-map.org) to determine the cellular localization of PGC-1α and its putative targets in the adult mouse cerebellum (Figure 3). At low magnification, PGC-1α mRNA exhibited high expression in the Purkinje cell layer (PCL; arrowheads) and deep cerebellar nuclei. Higher magnification of PGC-1α images in the cerebellar cortex revealed high expression in the PCL, moderate expression in the molecular layer (ML; arrows), and non-specific background expression in the granule cell layer (GCL), consistent with published data on PGC-1α protein expression in this brain region (Cowell et al., 2007). All eight PGC-1α-dependent transcripts exhibited high expression in the PCL and deep nuclei. Pascin2, Idh3a, Nceh1, Pdha1, and Uqcrfs1 most closely resembled the overall spatial pattern of PGC-1α, while Cplx1, Nefh, and Phyh exhibited higher expression in the GCL (concave arrowheads).

### Region-specific loss of Cplx1 and Nefh protein expression in mice lacking PGC-1α

**Figure 4:**
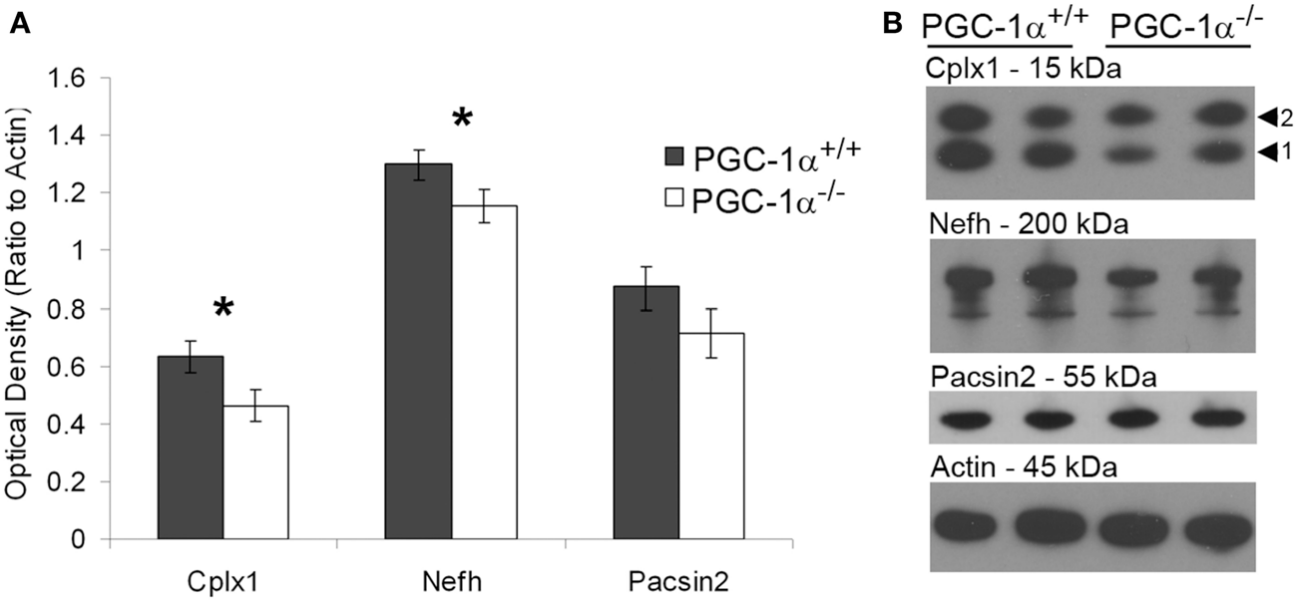
**Decreased protein expression of Nefh and Cplx1 in mice lacking PGC-1α**. Western blot analysis was performed on cerebellar homogenates of PGC-1α^+/+^ and ^−/−^ littermates at 30 days of age to determine if protein of PGC-1α-dependent genes was downregulated in a similar manner as the transcript. Expression of Cplx1 and Nefh, but not Pacsin2, was significantly reduced in ^−/−^ compared to ^+/+^ animals **(A)**. Representative Western blots are shown in **(B)**. One-tailed *t*-tests. ^*^p < 0.05. *n* = 7/group. Data are presented as mean ± SEM.

To investigate the likelihood of these changes in transcription influencing cell function, we determined whether protein, as well as transcript, expression of putative downstream targets of PGC-1α was decreased in the cerebellum of PGC-1α^−/−^ animals. To initially quantify overall protein levels, we conducted Western blot analysis on cerebellar homogenates from +/+ and −/− animals at 1 month of age with commercially available antibodies validated in our laboratory (Lucas et al., 2014). Of the tested antibodies, only Cplx1, Nefh, and Pacsin2 produced specific bands at the correct molecular weight. Expression of Cplx1 [*t*_(12)_ = 2.32, *p* = 0.02] and Nefh [*t*_(12)_ = 2.16, *p* = 0.03], but not Pacsin2, was significantly decreased in PGC-1α^−/−^ compared to ^+/+^ littermates (Figure 4).

**Figure 5:**
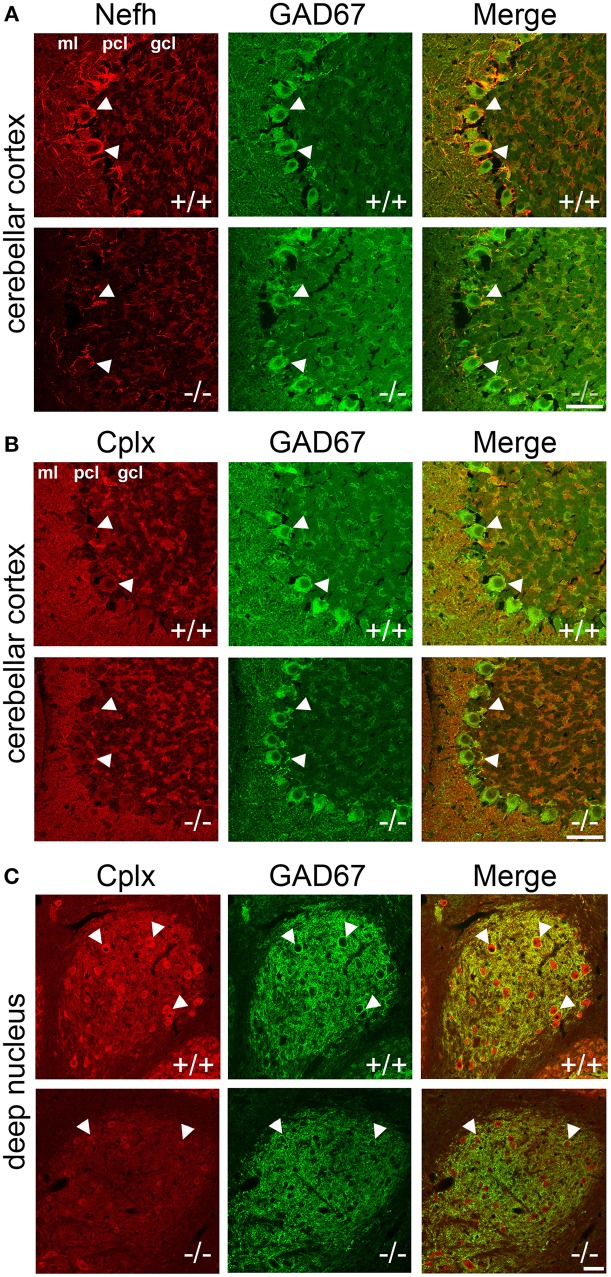
**Localization of Nefh and Cplx in PGC-1α ^−/−^ mice**. Confocal microscopy photos of immunofluorescence double-labeling of GAD67 with Nefh and Cplx1,2 reveals a differential reduction in the expression of these proteins in PGC-1α^−/−^ cerebellum. **(A)** Nefh exhibited high expression in Purkinje cell bodies (arrowheads) and dendritic processes in ^+/+^ mice. Somatic and dendritic expression was greatly reduced in the Purkinje cells of ^−/−^ animals. **(B)** Immunoreactivity for an antibody that recognizes both Cplx1 and Cplx2 did not reveal great expression for these proteins in Purkinje cell bodies (arrowheads), although some colocalization with GAD67-positive puncta surrounding Purkinje cells was observed in both ^+/+^ and ^−/−^ mice. **(C)** Cplx expression was of the greatest intensity in the deep cerebellar nuclei (interposed nucleus shown) in ^+/+^ animals. Cplx-positive cell bodies (arrowheads) were not GAD67-positive in the region, but Cplx exhibited a high degree of colocalization with GAD67-positive processes (note the high degree of GAD67 innervation of Cplx-positive cell bodies, presumably from Purkinje cell axon terminals). Cplx staining was greatly reduced in deep cerebellar nuclei of ^−/−^ animals. *n* = 4−5/group. Scale bars = 50 μm.

To localize the changes in protein expression to specific cell types, we conducted immunofluorescence colabeling of Nefh and Cplx with GAD67 in PGC-1α^+/+^ and ^−/−^ mice (Figure 5). In ^+/+^ animals, Nefh expression was highly concentrated in the PCL with clear labeling in Purkinje cell bodies and their dendritic trees extending into the ML. However, in ^−/−^ animals, Nefh labeling of Purkinje cells was greatly reduced, both in the soma and the dendrites (Figure 5A). Contradictory to transcript expression of Cplx1 (Figure 2B), labeling with an antibody that recognizes both Cplx1 and Cplx2 did not reveal appreciable staining of Cplx in the PCL. In both ^+/+^ and ^−/−^ animals, Cplx was colocalized to GAD67-positive puncta surrounding Purkinje cell bodies, and this expression did not appear to differ between ^+/+^ and ^−/−^ mice (Figure 5B). In the deep cerebellar nuclei, however, there was a high intensity of Cplx staining that was greatly reduced by deletion of PGC-1α (Figure 5C, interposed nucleus shown). Cplx-positive cell bodies were not GAD67-positive in this region, but Cplx exhibited a high degree of colocalization with GAD67-positive processes, presumably from Purkinje cell axon terminals. Cplx staining was greatly reduced in both the GAD67-positive processes and GAD67-negative cell bodies of deep cerebellar nuclei of ^−/−^ animals. Nefh expression, on the other hand, did not appear to differ between ^+/+^ and ^−/−^ mice in the deep cerebellar nuclei (data not shown).

### Conditional deletion of PGC-1α in PV-positive cells disrupts transcription of cerebellar PGC-1α-dependent genes

**Figure 6:**
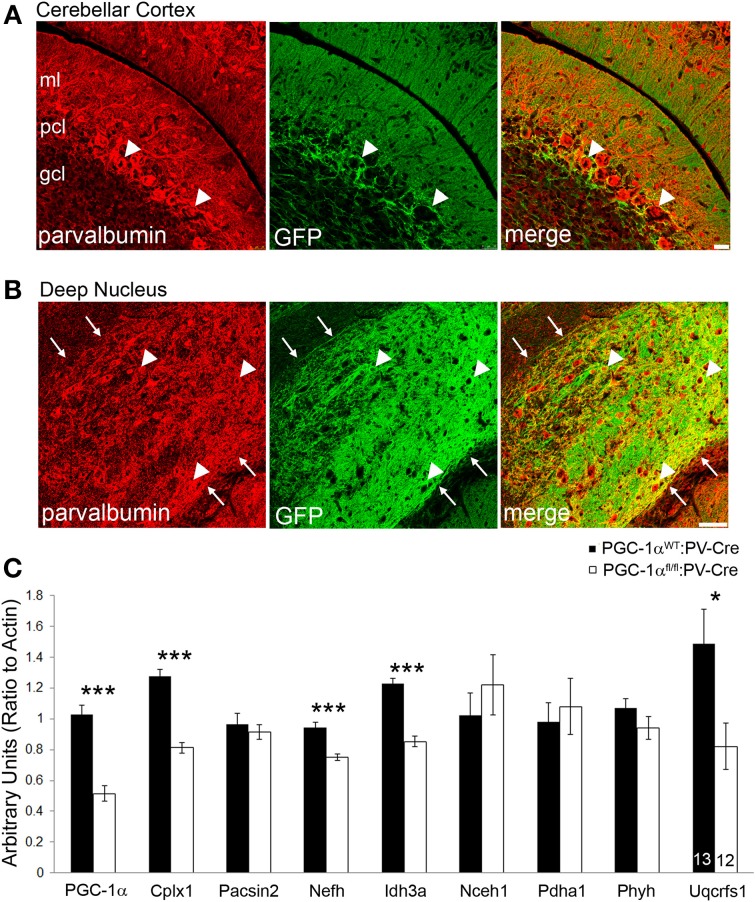
**Conditional deletion of PGC-1α in PV-positive cells decreases transcript expression of novel cerebellar PGC-1α-dependent genes**. To conditionally delete PGC-1α in a manner consistent with its reported expression (i.e., Purkinje cells, inhibitory interneurons of the cerebellar cortex, and cells of the deep cerebellar nuclei) PGC-1α floxed mice were crossed with mice expressing Cre recombinase driven by the parvalbumin promoter (PV-Cre). PV-Cre mice were crossed with mutant tomato/mutant green reporter mice to determine the specificity of Cre-mediated recombination at 3 months of age. PV expression is pseudocolored red from Cy5. **(A)** Green fluorescent protein (GFP) expression was high in the Purkinje cell layer (PCL; arrowheads), moderate in the molecular layer (ML), and low in the granule cell layer (GCL). Scale bar = 25 μm. **(B)** GFP expression was greatest in the deep cerebellar nuclei (interposed nucleus shown) and exhibited a high degree of colocalization to PV-positive terminals and, to a lesser extent, PV-positive cell bodies (arrowheads). Note the absence of GFP expression outside the borders of the nucleus (arrows). Scale bar = 75 μm. **(C)** Gene expression of PGC-1α and its putative targets was measured in cerebellar homogenates from 6-month-old PGC-1α^WT^ and ^fl/fl^ mice expressing PV-Cre by q-RT-PCR. Expression of PGC-1α, Cplx1, Nefh, Idh3a, and Uqcrfs1 were significantly reduced in PGC-1α^fl/fl^:PV-Cre mice. One-tailed *t*-tests. ^***^p < 0.0005, ^*^p < 0.05. n/group indicated on last bar histogram. Data presented as mean ± SEM.

Our immunofluorescence experiments suggest that the effects of PGC-1α ablation appear to be specific to different cellular populations, depending on the target transcript. However, the differential effects of loss of PGC-1α in distinct cellular populations are consistent with the previously published protein (Cowell et al., 2007) and mRNA (Figure 3) expression patterns demonstrating high levels of PGC-1α in Purkinje cells, interneurons in the ML, and cells of the deep cerebellar nuclei. Interestingly, all of these populations express high levels of the calcium binding protein parvalbumin (PV), so to test whether deletion of PGC-1α specifically within these neuronal populations could recapitulate the transcriptional deficiencies observed in the germline knockout animal, we crossed mice with LoxP sites flanking the 3–5 exon region of the PGC-1α gene (Lin et al., 2004) with mice expressing Cre recombinase driven by the PV promoter (Hippenmeyer et al., 2005). Cell-specific recombination was confirmed using reporter mice that express red fluorescent protein in the absence of recombination and green fluorescent protein (GFP) in the presence of recombination (Muzumdar et al., 2007). At 3 months of age, reporter mice exhibited GFP expression in the PCL and ML but not the GCL of the cerebellar cortex and in the deep cerebellar nuclei (Figures 6A,B), with the highest GFP expression in Purkinje cell bodies and axon terminals in the deep nuclei.

Gene expression was measured by q-RT-PCR in cerebellar homogenates from PGC-1α^WT^:PV-Cre and PGC-1α^fl/fl^:PV-Cre littermates at 6 months of age (Figure 6C). Conditional deletion of PGC-1α by PV-Cre resulted in a 50% decrease in the expression of PGC-1α [*t*_(23)_ = 7.69, *p* = 4.21 × 10^−8^] and also significantly reduced expression of Cplx1 [*t*_(23)_ = 8.57, *p* = 6.52 × 10^−9^], Nefh [*t*_(23)_ = 5.29, *p* = 1.34 × 10^−5^], Idh3a [*t*_(23)_ = 7.48, *p* = 6.68 × 10^−8^; Figure 6C], and Uqcrfs1 [*t*_(20)_ = 1.83, *p* = 0.04]. Interestingly, Pacsin2, **Nceh1**, Pdha1, and Phyh gene expression was unaffected by conditional deletion of PGC-1α, despite their apparent concentration in Purkinje cells (Figure 2). It is possible that Pacsin2, Nceh1, Pdha1, and Phyh are dependent on PGC-1α in PV-negative cell types or that cell-cell interactions in the complete knockout are required for decreases in their expression.

### PGC-1α ablation decreases purkinje cell viability and function

**Figure 7:**
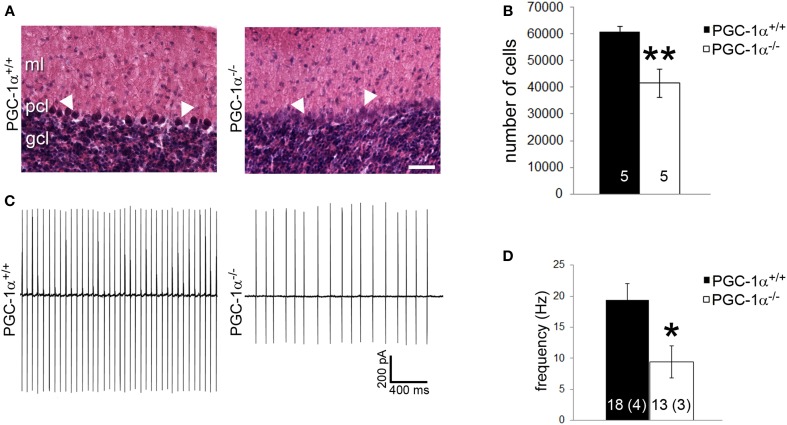
**PGC-1α ablation results in decreased Purkinje cell viability and function by 6 weeks of life. (A)** Stereological counts of Purkinje cells stained with hematoxylin and eosin (H&E) were conducted to obtain an unbiased estimate of the number of Purkinje cells. At 6 weeks of age, PGC-1α^−/−^ had significantly fewer Purkinje cells than ^+/+^ littermates. **(B)** Representative pictures of H&E stained Purkinje cells from 6-week-old animals. Note the deep staining in ^+/+^ animals compared to the often undetectable stain in the Purkinje cell layer (PCL) of ^−/−^ animals. GCL, granule cell layer. ML, molecular layer. Scale bar = 100 μm. **(C)** Loose patch electrophysiology was conducted in acute cerebellar slices to determine if loss of PGC-1α leads to Purkinje cell dysfunction. **(D)** Purkinje cell spike rate was significantly reduced in PGC-1α^−/−^ compared to ^+/+^ mice. Representative traces shown in **(C)** Two-tailed *t*-tests. ^**^p < 0.005, ^*^p < 0.05. n/group indicated on bar histograms. For electrophysiology data, the number of animals is indicated in parentheses after the number of cells. Data presented as mean ± SEM.

Purkinje cells exhibit a particularly high concentration of PGC-1α (Cowell et al., 2007), and our immunofluorescence and conditional knockout experiments suggest that Purkinje cells are dependent on PGC-1α for transcriptional regulation. Thus, we questioned whether the combined decrease of synaptic, structural, and metabolism-related genes caused by ablation of PGC-1α would lead to decreased neuronal viability and/or function in this cellular population. As a measure of Purkinje cell viability, we conducted stereological counts of H&E stained Purkinje cells in PGC-1α^+/+^ and ^−/−^ mice at 6 weeks of age, a time point after the onset of the previously described neurodegeneration in the striatum of this mouse line (Lucas et al., 2012). H&E labeling of Purkinje cells in PGC-1α^+/+^ animals exhibited a clear nuclear haematoxylin stain, while the haematoxylin stain of Purkinje cells in ^−/−^ mice was often very faint and cell bodies were at times undetectable between the ML and GCL (Figure 7A). Stereological estimation with the optical fractionator method demonstrated a ~30% reduction of the total number of Purkinje cells in ^−/−^ compared to ^+/+^ animals [*t*_(8)_ = 3.83, *p* = 0.005; Figure 7B].

Purkinje cells are one of the few neuronal populations that are spontaneously active in the absence of synaptic input (Hausser and Clark, 1997), allowing their firing rates to be measured in the absence of stimulation or drug application. As a measure of Purkinje cell basal function, we performed loose patch electrophysiology on Purkinje cells *in vitro* in acute cerebellar slices from PGC-1α^+/+^ and ^−/−^ animals at 6 weeks of age. Slices were perfused with oxygenated ACSF; once patched, Purkinje cells were allowed to acclimate to the pipette for 3 min to avoid events in response to mechanical stimulation. Spontaneous events were then recorded for 3 min with no stimulation (Figure 7C). We observed a 50% reduction in Purkinje cell spike rate in PGC-1α^−/−^ compared to ^+/+^ animals [*t*_(5)_ = 3.09, *p* = 0.03; Figure 7D].

## Discussion

In this manuscript, we present data showing that mice lacking PGC-1α exhibit a behavioral phenotype indicative of cerebellar dysfunction and show reduced cerebellar expression of transcripts involved in synaptic (Cplx1, Pacsin2), structural (Nefh), and metabolic (Idh3a, Nceh1, Pdha1, Phyh, and Uqcrfs1) functions. Using mice expressing Cre recombinase under the control of the PV promoter, we postnatally ablated PGC-1α in neuronal types enriched in its expression and observed reduced transcript expression of PGC-1α and its dependent genes Cplx1, Nefh, Idh3a, and Uqcrsf1, suggesting that PGC-1α regulation of these transcripts is specific to Purkinje cells, interneurons of the cerebellar cortex, and neurons of the deep cerebellar nuclei (Supplementary Table 4). Recombination in our conditional knockout animals was highest in Purkinje cells, and germline deletion of PGC-1α resulted in Purkinje cell loss and physiological dysfunction.

As the novel cerebellar PGC-1α-dependent transcripts are both upregulated by PGC-1α overexpression (*in vitro* data from Lucas et al., 2014) and reduced by its deletion (*in vivo* data from Figure 2), we hypothesize that Cplx1, Pacsin2, Nefh, Idh3a, Nceh1, Pdha1, Phyh, and Uqcrfs1 are all directly regulated by PGC-1α. To definitively determine whether these transcripts are direct targets of PGC-1α, chromatin immunoprecipitation assays are required; however, considering the cell type-specific differences in vulnerability to PGC-1α loss, these experiments are impractical. An alternative approach would be to identify the common intermediate transcription factors that PGC-1α interacts with to drive their expression. It is possible that genes within certain functional categories (neurotransmitter release, axonal structural support, and metabolism) are driven by PGC-1α's interaction with distinct sets of transcriptional regulators; for example, transcription of metabolic genes may be driven by the PGC-1α-interacting factor nuclear respiratory factor 1 (Wu et al., 1999). Further elucidation of the PGC-1α interactome may clarify the mechanisms by which PGC-1α can regulate parallel programs for functional, structural, and metabolic maturation.

Cplx1 and Nefh, but not Pascin2, were also decreased at the protein level in the cerebellum of PGC-1α^−/−^ mice. When we investigated the cellular distribution of protein expression, we found that Nefh was decreased in Purkinje cell bodies and dendritic processes, while Cplx was decreased in the deep cerebellar nuclei. Contradictory to the transcript pattern of Cplx1, very little Cplx protein was observed in the PCL. It is possible that the reduction in Cplx protein expression that was observed in the deep cerebellar nuclei was due to a loss of this synaptic protein in the axonal projections of Purkinje cells, whose sole synaptic targets are the deep cerebellar nuclei. However, we did not observe changes in Nefh expression in the white matter tracts from the Purkinje cells to the deep cerebellar nuclei, suggesting that the Purkinje cell axons themselves are still present. At the synapse, Cplx1 mediates synchronous neurotransmitter release by clamping the vesicle to the presynaptic membrane in a primed but unfused manner, thus controlling the timing of vesicular exocytosis (Sudhof and Rothman, 2009). Future experiments should be conducted to determine whether neurotransmitter release from Purkinje cells to the deep cerebellar nuclei is asynchronous, as abnormalities in synchronous neurotransmitter release were observed in the motor cortex of PGC-1α^fl/fl^:PV-Cre mice (Lucas et al., 2014).

While it has been established that loss of PGC-1α leads to vacuolization in motor regions of the forebrain (Lin et al., 2004; Leone et al., 2005; Lucas et al., 2012), no investigations of PGC-1α loss on cell viability have been conducted in the cerebellum to date. We here show a ~30% reduction in Purkinje cell number in PGC-1α^−/−^ mice, indicating that Purkinje cells are vulnerable to cell death in the absence of PGC-1α. Of the novel PGC-1α-dependent genes identified in this manuscript, Phyh is the only gene that has been shown to influence Purkinje cell viability. Mutations in the Phyh gene result in an autosomal recessive lipid storage disorder known as Refsum disease (Jansen et al., 1997). Clinically, patients present with severe cerebellar ataxia (Wierzbicki, 2007), and loss of Purkinje cells accompanied by ataxia has been reported in a Phyh ^−/−^ mouse model of this disease (Ferdinandusse et al., 2008).

While decreased Phyh expression is the most direct link between loss of PGC-1α and reduced Purkinje cell viability, it is also possible that compromised metabolism contributes to the observed cell loss. We previously found that the mitochondrial fusion gene mitofusin 2 (Mfn2) and the antioxidant gene manganese superoxide dismutase (MnSOD), both well-established targets of PGC-1α, were significantly reduced in cerebellar homogenates from PGC-1α^−/−^ mice (Lucas et al., 2010). Here, we reported that Uqcrfs1 is also a cerebellar PGC-1α-dependent gene. Uqcrfs1 is a component of complex III of the mitochondrial electron transport chain, and while other members of complex III can associate to form a complex in the absence of uqcrfs1, the resultant complex has limited enzymatic activity, leading to the degradation of complexes I and IV (Diaz et al., 2012). Thus, the combined decreases in expression of the metabolism-related genes Phyh, Idh3a, Mfn2, MnSOD, and Uqcrfs1 may lead to abnormalities in energy homeostasis or the increased production of reactive oxygen species, contributing to decreased viability of Purkinje cells of PGC-1α^−/−^ animals. Reduced energy production could also explain the decrease in spike rate, as none of the other PGC-1α-dependent genes identified to date, alone or in combination, have been reported to produce this physiological abnormality. Of note, cortical PV-positive interneurons also exhibit a decrease in firing rate in PGC-1α^−/−^ mice (Dougherty et al., 2014).

We previously reported that, in contrast to the cerebrum, PV expression in the cerebellum is not dependent on the presence of PGC-1α (Lucas et al., 2010). Here, no changes in the expression of synaptotagmin 2 (Syt2) were observed in the cerebellum of PGC-1α^−/−^ mice (Supplementary Table 3), while Syt2 is reduced by ~80% in the cortex of these mice (Lucas et al., 2014). This suggests that the mechanisms of PGC-1α-mediated gene regulation differ by region and cell type in the brain. Since PGC-1α's gene targets are defined by the transcription factors with which it interacts, it is likely that a different complement of factors interact with PGC-1α in cortical neurons than in cerebellar neurons. Multiple PGC-1α-interacting factors such as CREB, myocyte enhancing factor 2, and thyroid hormone receptor are expressed highly in the cerebellum; identification of the predominant factors that interact with PGC-1α in individual cell types would assist in predicting the specific subset of transcripts that would be affected by PGC-1α deficiency.

Regarding our observation of ataxia in PGC-1α^−/−^ mice, it is not currently clear which cell populations are contributing to this phenotype. A recent publication suggests that gait abnormalities in PGC-1α^−/−^ mice are more consistent with Purkinje cell dysfunction than dysfunction of granule cells or molecular layer interneurons (Vinueza Veloz et al., 2014). However, we did not perform CatWalk analysis on PGC-1α^fl/fl^:PV-Cre mice because our previous study did not reveal deficits in locomotor activity or motor coordination in these animals (Lucas et al., 2014). It is possible that the ataxia phenotype arises from cells that do not express PV, such as granule cells. As granule cell loss has been reported in Cplx1 ^−/−^ mice (Kielar et al., 2012), which exhibit profound ataxia (Glynn et al., 2005), PGC-1α may play an important role in regulation of Cplx1 and other dependent genes in granule cells. Other cells that could contribute to the ataxia phenotype could be motor neurons; it has not been shown whether motor neurons express PGC-1α, although overexpression of PGC-1α is capable of slowing disease progression in a mouse model of amyotrophic lateral sclerosis (Liang et al., 2011). A preliminary analysis of PGC-1α mRNA localization in the spinal cord using Allen Brain Atlas shows that PGC-1α mRNA is present in a subset of large neurons in the ventral gray matter of the spinal cord; gene expression loss, especially of Nefh which is highly concentrated in those cells, could be contributing to some of the motor deficits. Muscle cells are also affected by a lack of PGC-1α, although mice with muscle-specific deletion show only subtle motor impairments (Handschin et al., 2007).

It is also important to consider age differences when interpreting our behavioral data (conducted at 6 months of age) in light of our molecular findings (conducted at 4–6 weeks of age). Due to significantly decreased weight in PGC-1α^−/−^ mice at younger ages, gait analysis was conducted on mice at 6 months of age, when weight does not differ from PGC-1α^+/+^ mice, as differences in weight between genotypes can bias paw print detection with the automated Catwalk program. While we previously observed that striatal vacuolization and impaired rotarod performance does not significantly improve or decline between 1 and 3 months of age (Lucas et al., 2012), it is possible, given the additional metabolic targets in the cerebellum compared to the forebrain, that gait abnormalities are more pronounced at 6 months than earlier ages. While we did not perform transcriptional analysis from cerebella of 6 month-old PGC-1a −/− mice, we would predict that the putative targets that are reduced at 30 days of age would still be reduced at later ages (as demonstrated for the striatum; Lucas et al., 2012), but that other “off-target” transcripts may also be affected, due to compensatory mechanisms. Future studies will evaluate balance and gait in a cohort of younger animals with semi-quantitative approaches (Guyenet et al., 2010).

Recently, PGC-1α was also found to be involved in the pathology of Friedreich Ataxia. Friedreich Ataxia is an autosomal recessive disorder and is the most common of the early-onset hereditary ataxias (Pandolfo, 2009). Friedreich Ataxia is caused by an expanded GAA triplet repeat in the first intron of the frataxin gene, resulting in a 65–95% reduction in frataxin expression in Friedreich Ataxia patients (Campuzano et al., 1996). Transcript expression of PGC-1α and its downstream target MnSOD was found to be decreased in fibroblasts and lymphoblasts of patients with Friedreich Ataxia (Coppola et al., 2009; Marmolino et al., 2010). In fact, transcript expression of frataxin is highly correlated with that of PGC-1α in Friedreich Ataxia and control cells, and knock down of frataxin in cell culture also decreases transcript expression of PGC-1α (Coppola et al., 2009).

Related to the potential roles for PGC-1α in cerebellar gene dysregulation in other movement disorders, we have recently observed that the firing rate of Purkinje cells is reduced in transgenic and knock-in mouse models of Huntington Disease (Dougherty et al., 2012, 2013). Due to the interconnectivity between the basal ganglia and cerebellum and their joint roles in motor coordination and gait control, it is possible that cerebellar deficits contribute to the motor symptoms in Parkinson Disease as well (Wu and Hallett, 2013). Of note, the kinematic ataxia phenotype observed in PGC-1α^−/−^ mice (Figure 1) closely resembles the gait abnormalities reported in rodent models of both Huntington (Vandeputte et al., 2010) and Parkinson (Vlamings et al., 2007; Chuang et al., 2010) Diseases. Further experiments are required to determine whether dysregulation of PGC-1α-dependent programs of gene expression underlies some of the cell- and circuit-specific vulnerability in these disorders.

## Conflict of interest statement

The authors declare that the research was conducted in the absence of any commercial or financial relationships that could be construed as a potential conflict of interest.
