# Supplementary material for: Cerebellar transcriptional alterations with Purkinje cell dysfunction and loss in mice lacking PGC-1α
Source: Front Cell Neurosci. 2015 Jan 6;8:441. doi: 10.3389/fncel.2014.00441 (PMC4285109; doi:10.3389/fncel.2014.00441)
Supplement: Supplementary file 1 [file Table1.PDF]

Supplementary Table 1. *Applied Biosystems (ABI) primers with catalogue numbers.*

Gene Name	ABI#
ACSL6	Mm00522786_m1
AK1	Mm00445475_m1
ATP5A1	Mm00431960_m1
ATP5H	Mm02392026_g1
ATP5O	Mm01611862_g1
CDC42EP1	Mm00840486_m1
COX6C	Mm00835813_g1
COX7B	Mm00835076_g1
CPLX1	Mm00514378_m1
GAS6	Mm00490378_m1
GRIN2C	Mm00439180_m1
IDH3A	Mm00499674_m1
IMPA1	Mm00497770_m1
INPP5J	Mm00552486_m1
ITGB1BP1	Mm00492707_m1
KCNK1	Mm00434624_m1
LIFR	Mm00442942_m1
MT3	Mm00496661_g1
MYBPC3	Mm00435104_m1
NCEH1	Mm00626772_m1
NDUFS8	Mm00523063_m1
NEFH	Mm01191456_m1
OAF	Mm00618221_m1
PACSIN2	Mm00449303_m1
PDHA1	Mm00468675_m1
PHYH	Mm00477734_m1
PVALB	Mm00443100_m1
SERPINA5	Mm00435511_m1
SLC39A14	Mm01317439_m1
SPARCL1	Mm00447780_m1
ST8SIA1	Mm00456915_m1
ST8SIA5	Mm00457285_m1
STAC2	Mm00524631_m1
SYT2	Mm00436864_m1
UQCRFS1	Mm00481849_m1
UQCRH	Mm00835199_g1
VAMP2	Mm00494119_g1
