# Supplementary material for: Cerebellar transcriptional alterations with Purkinje cell dysfunction and loss in mice lacking PGC-1α
Source: Front Cell Neurosci. 2015 Jan 6;8:441. doi: 10.3389/fncel.2014.00441 (PMC4285109; doi:10.3389/fncel.2014.00441)
Supplement: Supplementary file 2 [file Table2.PDF]

Supplementary Table 2. *List of transcripts that overlapped with the* ppargc1a *or* pvalb *Neuroblast datasets not tested in cerebellar homogenates.*

Neuroblast	Gene Name
ppargc1a	CENTA1
	PFKFB2
	MTMR7
	ASNS
	PNPO
	CABLES2
	NDRG3
	CSDC2
	OSBPL6
	FBXO44
	EPS15L1
pvalb	METRNL
	SNRK
	STEAP2
	RPS6KA1
	DUSP4
	TMEM145
	GIT2
	HBEGF
