# Supplementary material for: Cerebellar transcriptional alterations with Purkinje cell dysfunction and loss in mice lacking PGC-1α
Source: Front Cell Neurosci. 2015 Jan 6;8:441. doi: 10.3389/fncel.2014.00441 (PMC4285109; doi:10.3389/fncel.2014.00441)
Supplement: Supplementary file 3 [file Table3.PDF]

Supplementary Table 3. *List and functions of transcripts measured in PGC-1α $^{-/-}$ cerebellum.* An unbiased microarray was performed to identify transcripts significantly upregulated by PGC-1α overexpression in SH-Sy5Y neuroblastoma cells (Lucas et al., 2014). Of the 27,000 tested transcripts, 1,067 were significantly upregulated by PGC-1α after FDR adjustment. Microarray data were then mined to test in cerebellar homogenates from PGC-1α $^{+/+}$, $^{+/-}$, and $^{-/-}$ mice based on their neuroanatomical overlap with PGC-1α or PV as determined by the Allen Brain Atlas feature Neuroblast. Superscript behind gene name indicates rationale for measuring: (1) top 10 most upregulated transcripts by PGC-1α with a murine homologue, (2) *ppargc1a* Neuroblast, and (3) *pvalb* Neuroblast. *Expression of transcripts measured in cerebellar homogenates are presented as the ratio of PGC-1α $^{-/-}$ to PGC-1α $^{+/+}$ animals (fold control). Gray highlight indicates transcripts that were significantly reduced in PGC-1α $^{-/-}$ cerebellum.

Gene Name	Cerebellum*	Function
ACSL6 [2]	0.83 ± 0.09	Lipid metabolism
AK1 [2]	0.91 ± 0.09	Adenine nucleotide metabolism
ATP5A1 [2]	0.93 ± 0.05	ATP synthesis
ATP5H [2]	1.06 ± 0.19	ATP synthesis
ATP5O [2]	0.93 ± 0.14	ATP synthesis
CDC42EP1 [3]	0.81 ± 0.05	Cytoskeleton reorganization
COX6C [2]	0.81 ± 0.16	Electron transport (complex IV)
COX7B [2]	0.78 ± 0.13	Electron transport
CPLX1 [1,2]	0.54 ± 0.11	Neurotransmitter release
GAS6 [2]	0.87 ± 0.09	Cell proliferation
GRIN2C [3]	1.17 ± 0.29	Excitatory transmission
IDH3A [1]	0.74± 0.07	TCA cycle
IMPA1 [2]	0.99 ± 0.08	Myoinositol metabolism
INPP5J [2]	0.90 ± 0.13	Phosphatidylinositol met.
ITGB1BP1 [3]	0.93 ± 0.14	Cell adhesion
KCNK1 [2,3]	1.10 ± 0.14	Inward rectifying K^+ channel
LIFR [2]	0.85 ± 0.07	Leukemia inhib. factor binding
MT3 [1]	0.83 ± 0.13	Antioxidant, metal binding
MYBPC3 [1]	1.20 ± 0.16	Myosin binding
NCEH1 [2]	0.74 ± 0.04	Lipid metabolism
NDUFS8 [2]	0.85 ± 0.11	Respiration (complex I)
NEFH [3]	0.54 ± 0.07	Structural support
OAF [1]	1.04± 0.14	Unknown
PACSIN2 [2]	0.68 ± 0.05	Vesicle trafficking
PDHA1 [2]	0.70 ± 0.04	Glycolysis, TCA cycle
PHYH [2]	0.55 ± 0.03	Lipid metabolism
PVALB [1,2]	0.81 ± 0.10	Calcium buffer
SERPINA5 [1]	*not detectable*	Proteolysis and lipid transport
SLC39A14 [3]	0.89 ± 0.15	Zinc transport
SPARCL1 [3]	1.02 ± 0.12	Extracellular matrix
ST8SIA1 [2]	0.94 ± 0.18	Ganglioside metabolism
ST8SIA5 [2]	0.94 ± 0.18	Carbohydrate metabolism
STAC2 [3]	0.76 ± 0.14	Metal ion binding
SYT2 [3]	1.22 ± 0.14	Neurotransmitter release
UQCRFS1 [2]	0.65 ± 0.09	Respiration (complex III)
UQCRH [2]	1.07 ± 0.24	Electron transport
VAMP2 [2]	1.07 ± 0.10	Neurotransmitter release
