# Supplementary material for: Cerebellar transcriptional alterations with Purkinje cell dysfunction and loss in mice lacking PGC-1α
Source: Front Cell Neurosci. 2015 Jan 6;8:441. doi: 10.3389/fncel.2014.00441 (PMC4285109; doi:10.3389/fncel.2014.00441)
Supplement: Supplementary file 4 [file Table4.PDF]

Function	Name	Transcript Expression (-/- versus +/+ cerebellar homogenate)	Protein Expression	Localization of Protein Loss (-/- versus +/+ immunofluoresence)	Transcript Expression in PV+ Cells (WT:PV-Cre versus Fl/Fl:PV-Cre)
Synaptic	Cplx1	decreased	decreased	Purkinje cell terminals in deep nuclei	decreased
	Pacsin2	decreased	no difference	-	no difference
Structural	Nefh	decreased	decreased	Purkinje cell soma and dendrites	decreased
Metabolic	Idh3a	decreased	-	-	decreased
	Nceh1	decreased	-	-	decreased
	Pdha1	decreased	-	-	no difference
	Phyh	decreased	-	-	no difference
	Uqcrfs1	decreased	-	-	decreased

Supplementary Table 4. *Summary of results.* The novel PGC-1α-dependent transcripts Cplx1, Pacsin2, Nefh, Idh3a, Nceh1, Pdha1, Phyh, and Uqcrfs1 were significantly decreased in cerebellar homogenates from PGC-1α $^{-/-}$ compared to $^{+/+}$ animals. Due to primary antibody limitations, only Cplx1, Pacsin2, and Nefh were confirmed at the protein level with Western blot analysis; we observed significant reductions in Cplx1 and Nefh but not Pacsin2 in cerebellar homogenates from PGC-1α $^{-/-}$ compared to $^{+/+}$ animals. To determine cellular colocalization of protein loss, we conducted immunofluorescence staining and found compartmentalized loss of Cplx1 and Nefh expression. While Nefh was reduced in Purkinje cell bodies and dendrites, Cplx1 was reduced in Purkinje cell axon terminals in the deep cerebellar nuclei. To further elucidate cell-specific transcriptional regulation mediated by PGC-1α in the cerebellum, we conditionally deleted PGC-1α in parvalbumin (PV)-expressing cells, which includes Purkinje cells as well as interneurons and neurons of the deep cerebellar nuclei, using the LoxP/Cre system. Transcript expression of PGC-1α and its dependent genes Cplx1, Nefh, Idh3a, and Uqcrfs1 were significantly reduced in cerebellar homogenates from PGC-1α$^{fl/fl}$:PV-Cre compared to PGC-1α^{WT}:PV-Cre mice, suggesting that PGC-1α regulates expression of Pacsin2, Pdha1, and Phyh in non-PV-expressing cells, such as granule cells, non-PV+ interneurons, and glia.
Dashes (-) indicate assays that were not tested.
